# Bis(5-chloro­quinolin-8-olato-κ^2^ *N*,*O*)bis­(propan-2-olato-κ*O*)titanium(IV)

**DOI:** 10.1107/S1600536809004383

**Published:** 2009-02-13

**Authors:** Yousef Fazaeli, Ezzatollah Najafi, Mostafa M. Amini, Seik Weng Ng

**Affiliations:** a Department of Chemistry, General Campus, Shahid Beheshti University, Tehran 1983963113, Iran; b Department of Chemistry, University of Malaya, 50603 Kuala Lumpur, Malaysia

## Abstract

The Ti^IV^ atom in the title compound, [Ti(C_9_H_5_ClNO)_2_(C_3_H_7_O)_2_], is chelated by the substituted quinolin-8-olate anions in a distorted octa­hedral geometry. The N-donor atoms are in a *cis* alignment as are the O atoms of the propan-2-olate groups; the O atoms of the quinolin-8-olate groups are *trans* to each other. One C atom of one propan-2-olate group is disordered over two positions with occupancies of 0.733 (8):0.267 (8).

## Related literature

For diisoproxidobis(quinolin-8-olato)titanium(IV), see: Zeng *et al.* (2002 ▶). For diisopropoxidobis(2-methyl­quinolin-8-olato)titanium(IV), see: Faza­eli *et al.* (2008 ▶).
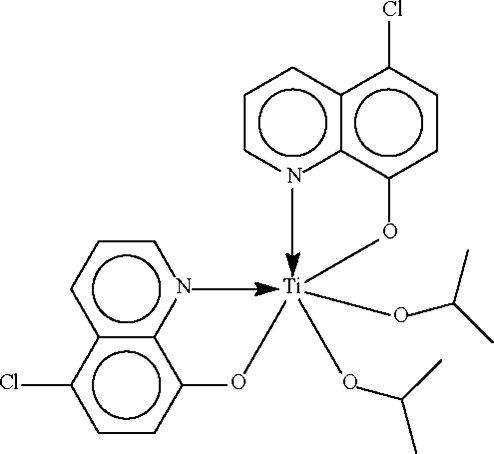


## Experimental

### 

#### Crystal data

[Ti(C_9_H_5_ClNO)_2_(C_3_H_7_O)_2_]
*M* *_r_* = 523.25
Triclinic, 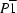

*a* = 8.2170 (2) Å
*b* = 12.1847 (3) Å
*c* = 13.8113 (3) Å
α = 109.555 (1)°
β = 105.090 (1)°
γ = 103.785 (1)°
*V* = 1174.89 (5) Å^3^
*Z* = 2
Mo *K*α radiation
μ = 0.63 mm^−1^
*T* = 100 (2) K
0.40 × 0.08 × 0.08 mm

#### Data collection

Bruker SMART APEX diffractometer
Absorption correction: multi-scan (*SADABS*; Sheldrick, 1996 ▶) *T*_min_ = 0.788, *T*_max_ = 0.952
9665 measured reflections
5285 independent reflections
4126 reflections with *I* > 2σ(*I*)
*R*_int_ = 0.024

#### Refinement

*R*[*F* ^2^ > 2σ(*F* ^2^)] = 0.039
*wR*(*F* ^2^) = 0.102
*S* = 1.04
5285 reflections
308 parameters
18 restraints
H-atom parameters constrained
Δρ_max_ = 0.35 e Å^−3^
Δρ_min_ = −0.32 e Å^−3^

### 

Data collection: *APEX2* (Bruker, 2008 ▶); cell refinement: *SAINT* (Bruker, 2008 ▶); data reduction: *SAINT*; program(s) used to solve structure: *SHELXS97* (Sheldrick, 2008 ▶); program(s) used to refine structure: *SHELXL97* (Sheldrick, 2008 ▶); molecular graphics: *X-SEED* (Barbour, 2001 ▶); software used to prepare material for publication: *publCIF* (Westrip, 2009 ▶).

## Supplementary Material

Crystal structure: contains datablocks global, I. DOI: 10.1107/S1600536809004383/bt2867sup1.cif

Structure factors: contains datablocks I. DOI: 10.1107/S1600536809004383/bt2867Isup2.hkl

Additional supplementary materials:  crystallographic information; 3D view; checkCIF report

## Figures and Tables

**Fig. 1.**
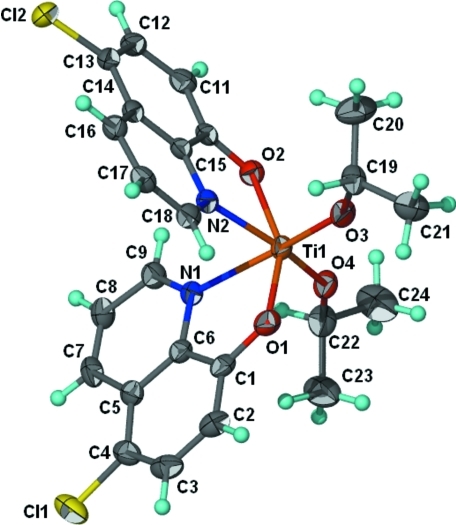
Anisotropic displacement ellipsoid plot (Barbour, 2001) of Ti(C3H7O)2(C9H5ClNO)2(C3H7O)2; ellipsoids are drawn at the 70% probability level and H atoms of arbitrary radius. Only the major occupied site of the disordered atom is shown.

## supplementary crystallographic information

### Experimental

5-Chloro-8-hydroxyquinoline (1.79 g, 10 mmol) was added to titanium isoproxide (2.84 g, 10 mmol) in toluene (20 ml).The mixture was stirred for a day and the solvent then removed under reduced pressure to furnish a deep yellow solid. The solid was crystallized from a dichloromethane/*n*-hexane to give yellow crystals, m.p. 439 K.

### Refinement

H-atoms were placed in calculated positions (C—H 0.93–0.99 Å) and were included in the refinement in the riding model approximation, with *U*(H) set to 1.2–1.5*U*(C).

The C19 atom is disordered over two positions. This was refined was two atoms, and the C–C distances were restrained to 1.54±0.01 Å. The O–C distances were restrained to 1.45 + 0.01 Å. The anisotropic displacement parameters of the two disordered atoms were restrained to be nearly isotropic. The disorder refined to a 0.733 (8):0.267 (8) ratio.

### Crystal data

**Table:**

[Ti(C_9_H_5_ClNO)_2_(C_3_H_7_O)_2_]	*Z* = 2
*M**_r_* = 523.25	*F*(000) = 540
Triclinic, *P*1	*D*_x_ = 1.479 Mg m^−^^3^
Hall symbol: -P 1	Mo *K*α radiation, λ = 0.71073 Å
*a* = 8.2170 (2) Å	Cell parameters from 2883 reflections
*b* = 12.1847 (3) Å	θ = 2.6–28.1°
*c* = 13.8113 (3) Å	µ = 0.63 mm^−^^1^
α = 109.555 (1)°	*T* = 100 K
β = 105.090 (1)°	Prism, yellow
γ = 103.785 (1)°	0.40 × 0.08 × 0.08 mm
*V* = 1174.89 (5) Å^3^	

### Data collection

**Table:**

Bruker SMART APEX diffractometer	5285 independent reflections
Radiation source: fine-focus sealed tube	4126 reflections with *I* > 2σ(*I*)
graphite	*R*_int_ = 0.024
ω scans	θ_max_ = 27.5°, θ_min_ = 1.7°
Absorption correction: multi-scan (*SADABS*; Sheldrick, 1996)	*h* = −10→10
*T*_min_ = 0.788, *T*_max_ = 0.952	*k* = −15→15
9665 measured reflections	*l* = −17→17

### Refinement

**Table:**

Refinement on *F*^2^	Primary atom site location: structure-invariant direct methods
Least-squares matrix: full	Secondary atom site location: difference Fourier map
*R*[*F*^2^ > 2σ(*F*^2^)] = 0.039	Hydrogen site location: inferred from neighbouring sites
*wR*(*F*^2^) = 0.102	H-atom parameters constrained
*S* = 1.04	*w* = 1/[σ^2^(*F*_o_^2^) + (0.0434*P*)^2^ + 0.5978*P*] where *P* = (*F*_o_^2^ + 2*F*_c_^2^)/3
5285 reflections	(Δ/σ)_max_ = 0.001
308 parameters	Δρ_max_ = 0.35 e Å^−^^3^
18 restraints	Δρ_min_ = −0.32 e Å^−^^3^

### Special details

**Table:**

Geometry. All e.s.d.'s (except the e.s.d. in the dihedral angle between two l.s. planes) are estimated using the full covariance matrix. The cell e.s.d.'s are taken into account individually in the estimation of e.s.d.'s in distances, angles and torsion angles; correlations between e.s.d.'s in cell parameters are only used when they are defined by crystal symmetry. An approximate (isotropic) treatment of cell e.s.d.'s is used for estimating e.s.d.'s involving l.s. planes.

### Fractional atomic coordinates and isotropic or equivalent isotropic displacement parameters (Å^2^)

**Table:**

	*x*	*y*	*z*	*U*_iso_*/*U*_eq_	Occ. (<1)
Ti1	0.36699 (5)	0.46368 (4)	0.21132 (3)	0.02005 (11)	
Cl1	−0.42848 (9)	0.06005 (6)	0.18350 (5)	0.03744 (17)	
Cl2	0.23370 (8)	1.01980 (5)	0.47176 (5)	0.02848 (15)	
O1	0.3245 (2)	0.35579 (15)	0.28814 (13)	0.0251 (4)	
O2	0.3248 (2)	0.59246 (14)	0.16226 (12)	0.0222 (3)	
O3	0.6063 (2)	0.52223 (15)	0.27602 (14)	0.0283 (4)	
O4	0.3252 (2)	0.34879 (15)	0.07709 (13)	0.0257 (4)	
N1	0.0664 (2)	0.40036 (17)	0.17023 (15)	0.0199 (4)	
N2	0.3560 (2)	0.60873 (17)	0.35960 (15)	0.0196 (4)	
C1	0.1581 (3)	0.2891 (2)	0.27005 (18)	0.0217 (5)	
C2	0.1141 (3)	0.1993 (2)	0.30843 (19)	0.0260 (5)	
H2	0.2072	0.1836	0.3529	0.031*	
C3	−0.0671 (4)	0.1311 (2)	0.28229 (19)	0.0286 (5)	
H3	−0.0945	0.0692	0.3089	0.034*	
C4	−0.2052 (3)	0.1516 (2)	0.21957 (19)	0.0259 (5)	
C5	−0.1694 (3)	0.2452 (2)	0.18031 (18)	0.0218 (5)	
C6	0.0136 (3)	0.3111 (2)	0.20600 (18)	0.0197 (4)	
C7	−0.2991 (3)	0.2793 (2)	0.11910 (19)	0.0259 (5)	
H7	−0.4240	0.2386	0.1009	0.031*	
C8	−0.2436 (3)	0.3709 (2)	0.08642 (19)	0.0260 (5)	
H8	−0.3296	0.3954	0.0465	0.031*	
C9	−0.0594 (3)	0.4286 (2)	0.11196 (19)	0.0233 (5)	
H9	−0.0231	0.4904	0.0865	0.028*	
C10	0.3085 (3)	0.6930 (2)	0.22925 (18)	0.0191 (4)	
C11	0.2780 (3)	0.7874 (2)	0.20186 (19)	0.0240 (5)	
H11	0.2710	0.7842	0.1311	0.029*	
C12	0.2573 (3)	0.8885 (2)	0.2785 (2)	0.0238 (5)	
H12	0.2338	0.9518	0.2578	0.029*	
C13	0.2702 (3)	0.8977 (2)	0.38176 (19)	0.0213 (5)	
C14	0.3095 (3)	0.8066 (2)	0.41672 (18)	0.0191 (4)	
C15	0.3243 (3)	0.7044 (2)	0.33780 (18)	0.0179 (4)	
C16	0.3405 (3)	0.8113 (2)	0.52348 (18)	0.0223 (5)	
H16	0.3349	0.8796	0.5798	0.027*	
C17	0.3787 (3)	0.7167 (2)	0.54536 (19)	0.0229 (5)	
H17	0.4029	0.7196	0.6175	0.027*	
C18	0.3819 (3)	0.6154 (2)	0.46011 (18)	0.0217 (5)	
H18	0.4036	0.5487	0.4754	0.026*	
C19	0.7545 (4)	0.6058 (3)	0.3799 (3)	0.0259 (11)	0.733 (8)
H19	0.7047	0.6190	0.4400	0.031*	0.733 (8)
C19'	0.7943 (8)	0.5966 (6)	0.3237 (6)	0.017 (2)	0.267 (8)
H19'	0.8420	0.5836	0.2628	0.020*	0.267 (8)
C20	0.8248 (4)	0.7275 (2)	0.3771 (3)	0.0434 (7)	
H20A	0.7250	0.7564	0.3565	0.065*	0.733 (8)
H20B	0.8825	0.7187	0.3225	0.065*	0.733 (8)
H20C	0.9135	0.7882	0.4503	0.065*	0.733 (8)
H20D	0.7137	0.7423	0.3483	0.065*	0.267 (8)
H20E	0.9214	0.7755	0.3621	0.065*	0.267 (8)
H20F	0.8600	0.7537	0.4571	0.065*	0.267 (8)
C21	0.8883 (3)	0.5458 (2)	0.4018 (2)	0.0340 (6)	
H21A	0.9928	0.6047	0.4691	0.051*	0.733 (8)
H21B	0.9275	0.5215	0.3390	0.051*	0.733 (8)
H21C	0.8333	0.4716	0.4116	0.051*	0.733 (8)
H21D	0.9897	0.6148	0.4651	0.051*	0.267 (8)
H21E	0.9331	0.4846	0.3617	0.051*	0.267 (8)
H21F	0.8019	0.5060	0.4280	0.051*	0.267 (8)
C22	0.1934 (3)	0.2575 (2)	−0.0269 (2)	0.0297 (5)	
H22	0.0879	0.2846	−0.0447	0.036*	
C23	0.1300 (4)	0.1341 (2)	−0.0203 (2)	0.0415 (7)	
H23A	0.0799	0.1439	0.0379	0.062*	
H23B	0.2322	0.1064	−0.0029	0.062*	
H23C	0.0368	0.0718	−0.0915	0.062*	
C24	0.2717 (4)	0.2478 (3)	−0.1150 (2)	0.0473 (8)	
H24A	0.3114	0.3294	−0.1168	0.071*	
H24B	0.1797	0.1868	−0.1871	0.071*	
H24C	0.3750	0.2210	−0.0987	0.071*	

### Atomic displacement parameters (Å^2^)

**Table:**

	*U*^11^	*U*^22^	*U*^33^	*U*^12^	*U*^13^	*U*^23^
Ti1	0.0143 (2)	0.0191 (2)	0.0242 (2)	0.00694 (16)	0.00535 (16)	0.00694 (17)
Cl1	0.0332 (4)	0.0336 (4)	0.0379 (4)	−0.0009 (3)	0.0177 (3)	0.0117 (3)
Cl2	0.0310 (3)	0.0203 (3)	0.0383 (3)	0.0130 (2)	0.0161 (3)	0.0121 (3)
O1	0.0194 (8)	0.0224 (8)	0.0315 (9)	0.0097 (7)	0.0038 (7)	0.0118 (7)
O2	0.0233 (8)	0.0226 (8)	0.0226 (8)	0.0087 (7)	0.0102 (7)	0.0100 (7)
O3	0.0164 (8)	0.0224 (9)	0.0354 (10)	0.0067 (7)	0.0038 (7)	0.0045 (7)
O4	0.0188 (8)	0.0233 (8)	0.0267 (8)	0.0074 (7)	0.0057 (7)	0.0033 (7)
N1	0.0181 (10)	0.0209 (10)	0.0229 (9)	0.0088 (8)	0.0078 (8)	0.0103 (8)
N2	0.0147 (9)	0.0205 (10)	0.0244 (10)	0.0069 (8)	0.0063 (8)	0.0107 (8)
C1	0.0251 (12)	0.0156 (11)	0.0212 (11)	0.0084 (9)	0.0077 (9)	0.0042 (9)
C2	0.0327 (14)	0.0216 (12)	0.0229 (12)	0.0118 (10)	0.0072 (10)	0.0093 (10)
C3	0.0412 (15)	0.0214 (12)	0.0250 (12)	0.0093 (11)	0.0147 (11)	0.0115 (10)
C4	0.0284 (13)	0.0206 (12)	0.0238 (12)	0.0027 (10)	0.0133 (10)	0.0053 (10)
C5	0.0238 (12)	0.0210 (11)	0.0207 (11)	0.0080 (10)	0.0114 (9)	0.0064 (9)
C6	0.0209 (11)	0.0176 (11)	0.0197 (11)	0.0080 (9)	0.0076 (9)	0.0062 (9)
C7	0.0165 (11)	0.0302 (13)	0.0251 (12)	0.0066 (10)	0.0076 (10)	0.0062 (10)
C8	0.0199 (12)	0.0335 (13)	0.0262 (12)	0.0124 (10)	0.0079 (10)	0.0130 (11)
C9	0.0210 (12)	0.0270 (12)	0.0257 (12)	0.0103 (10)	0.0086 (10)	0.0145 (10)
C10	0.0140 (10)	0.0195 (11)	0.0234 (11)	0.0039 (9)	0.0071 (9)	0.0100 (9)
C11	0.0216 (12)	0.0262 (12)	0.0266 (12)	0.0065 (10)	0.0088 (10)	0.0154 (10)
C12	0.0206 (12)	0.0228 (12)	0.0330 (13)	0.0089 (10)	0.0094 (10)	0.0171 (10)
C13	0.0174 (11)	0.0169 (11)	0.0284 (12)	0.0059 (9)	0.0087 (9)	0.0081 (9)
C14	0.0134 (10)	0.0181 (11)	0.0247 (11)	0.0049 (9)	0.0061 (9)	0.0088 (9)
C15	0.0137 (10)	0.0190 (11)	0.0228 (11)	0.0050 (9)	0.0068 (9)	0.0114 (9)
C16	0.0199 (11)	0.0212 (11)	0.0231 (11)	0.0060 (9)	0.0076 (9)	0.0074 (9)
C17	0.0200 (11)	0.0283 (12)	0.0224 (11)	0.0071 (10)	0.0084 (9)	0.0139 (10)
C18	0.0186 (11)	0.0244 (12)	0.0252 (12)	0.0092 (9)	0.0066 (9)	0.0143 (10)
C19	0.0175 (17)	0.0293 (18)	0.0232 (19)	0.0073 (14)	0.0032 (14)	0.0064 (14)
C19'	0.013 (4)	0.026 (4)	0.015 (4)	0.006 (3)	0.010 (3)	0.008 (3)
C20	0.0372 (16)	0.0251 (14)	0.0494 (17)	0.0050 (12)	−0.0017 (13)	0.0123 (13)
C21	0.0277 (14)	0.0329 (14)	0.0380 (15)	0.0077 (11)	0.0046 (11)	0.0190 (12)
C22	0.0214 (12)	0.0307 (13)	0.0245 (12)	0.0076 (11)	0.0023 (10)	0.0034 (10)
C23	0.0440 (17)	0.0293 (14)	0.0328 (14)	−0.0005 (12)	0.0151 (13)	0.0006 (12)
C24	0.0478 (18)	0.0468 (18)	0.0278 (14)	0.0001 (14)	0.0102 (13)	0.0080 (13)

### Geometric parameters (Å, °)

**Table:**

Ti1—O3	1.7788 (16)	C12—H12	0.9500
Ti1—O4	1.7936 (16)	C13—C14	1.420 (3)
Ti1—O1	1.9707 (16)	C14—C16	1.408 (3)
Ti1—O2	1.9723 (16)	C14—C15	1.410 (3)
Ti1—N2	2.2527 (18)	C16—C17	1.369 (3)
Ti1—N1	2.2554 (18)	C16—H16	0.9500
Cl1—C4	1.742 (2)	C17—C18	1.403 (3)
Cl2—C13	1.738 (2)	C17—H17	0.9500
O1—C1	1.323 (3)	C18—H18	0.9500
O2—C10	1.327 (3)	C19—C20	1.475 (4)
O3—C19'	1.430 (6)	C19—C21	1.481 (4)
O3—C19	1.449 (3)	C19—H19	1.0000
O4—C22	1.421 (3)	C19'—C20	1.441 (7)
N1—C9	1.325 (3)	C19'—C21	1.543 (6)
N1—C6	1.365 (3)	C19'—H19'	1.0000
N2—C18	1.319 (3)	C20—H20A	0.9800
N2—C15	1.362 (3)	C20—H20B	0.9800
C1—C2	1.381 (3)	C20—H20C	0.9800
C1—C6	1.424 (3)	C20—H20D	0.9800
C2—C3	1.402 (4)	C20—H20E	0.9800
C2—H2	0.9500	C20—H20F	0.9800
C3—C4	1.367 (3)	C21—H21A	0.9800
C3—H3	0.9500	C21—H21B	0.9800
C4—C5	1.421 (3)	C21—H21C	0.9800
C5—C6	1.410 (3)	C21—H21D	0.9800
C5—C7	1.414 (3)	C21—H21E	0.9800
C7—C8	1.364 (3)	C21—H21F	0.9800
C7—H7	0.9500	C22—C24	1.503 (4)
C8—C9	1.400 (3)	C22—C23	1.512 (4)
C8—H8	0.9500	C22—H22	1.0000
C9—H9	0.9500	C23—H23A	0.9800
C10—C11	1.381 (3)	C23—H23B	0.9800
C10—C15	1.427 (3)	C23—H23C	0.9800
C11—C12	1.410 (3)	C24—H24A	0.9800
C11—H11	0.9500	C24—H24B	0.9800
C12—C13	1.366 (3)	C24—H24C	0.9800
			
O3—Ti1—O4	103.15 (7)	O3—C19—H19	107.6
O3—Ti1—O1	95.29 (7)	C20—C19—H19	107.6
O4—Ti1—O1	100.15 (7)	C21—C19—H19	107.6
O3—Ti1—O2	101.90 (7)	O3—C19'—C20	111.7 (5)
O4—Ti1—O2	96.17 (7)	O3—C19'—C21	107.1 (4)
O1—Ti1—O2	152.90 (7)	C20—C19'—C21	113.8 (5)
O3—Ti1—N2	89.20 (7)	O3—C19'—H19'	108.0
O4—Ti1—N2	166.55 (7)	C20—C19'—H19'	108.0
O1—Ti1—N2	83.74 (7)	C21—C19'—H19'	108.0
O2—Ti1—N2	75.79 (6)	C19'—C20—C19	36.0 (3)
O3—Ti1—N1	166.54 (7)	C19'—C20—H20A	118.7
O4—Ti1—N1	88.50 (7)	C19—C20—H20A	109.5
O1—Ti1—N1	75.78 (6)	C19'—C20—H20B	73.8
O2—Ti1—N1	83.22 (7)	C19—C20—H20B	109.5
N2—Ti1—N1	79.93 (7)	H20A—C20—H20B	109.5
C1—O1—Ti1	120.40 (14)	C19'—C20—H20C	127.4
C10—O2—Ti1	120.95 (13)	C19—C20—H20C	109.5
C19'—O3—C19	36.5 (3)	H20A—C20—H20C	109.5
C19'—O3—Ti1	165.3 (3)	H20B—C20—H20C	109.5
C19—O3—Ti1	142.82 (19)	C19'—C20—H20D	109.5
C22—O4—Ti1	146.48 (15)	C19—C20—H20D	102.1
C9—N1—C6	118.54 (19)	H20A—C20—H20D	9.2
C9—N1—Ti1	130.57 (15)	H20B—C20—H20D	107.9
C6—N1—Ti1	110.63 (14)	H20C—C20—H20D	118.0
C18—N2—C15	118.38 (19)	C19'—C20—H20E	109.5
C18—N2—Ti1	130.30 (15)	C19—C20—H20E	141.0
C15—N2—Ti1	111.26 (14)	H20A—C20—H20E	104.6
O1—C1—C2	124.8 (2)	H20B—C20—H20E	39.1
O1—C1—C6	117.6 (2)	H20C—C20—H20E	75.3
C2—C1—C6	117.6 (2)	H20D—C20—H20E	109.5
C1—C2—C3	120.5 (2)	C19'—C20—H20F	109.5
C1—C2—H2	119.8	C19—C20—H20F	79.8
C3—C2—H2	119.8	H20A—C20—H20F	104.7
C4—C3—C2	121.7 (2)	H20B—C20—H20F	138.4
C4—C3—H3	119.2	H20C—C20—H20F	34.5
C2—C3—H3	119.2	H20D—C20—H20F	109.5
C3—C4—C5	120.7 (2)	H20E—C20—H20F	109.5
C3—C4—Cl1	120.28 (19)	C19—C21—C19'	34.6 (3)
C5—C4—Cl1	118.97 (19)	C19—C21—H21A	109.5
C6—C5—C7	117.1 (2)	C19'—C21—H21A	118.5
C6—C5—C4	116.6 (2)	C19—C21—H21B	109.5
C7—C5—C4	126.4 (2)	C19'—C21—H21B	75.1
N1—C6—C5	122.5 (2)	H21A—C21—H21B	109.5
N1—C6—C1	114.56 (19)	C19—C21—H21C	109.5
C5—C6—C1	122.9 (2)	C19'—C21—H21C	127.0
C8—C7—C5	119.6 (2)	H21A—C21—H21C	109.5
C8—C7—H7	120.2	H21B—C21—H21C	109.5
C5—C7—H7	120.2	C19—C21—H21D	102.1
C7—C8—C9	119.8 (2)	C19'—C21—H21D	109.5
C7—C8—H8	120.1	H21A—C21—H21D	9.1
C9—C8—H8	120.1	H21B—C21—H21D	108.2
N1—C9—C8	122.5 (2)	H21C—C21—H21D	117.7
N1—C9—H9	118.8	C19—C21—H21E	140.1
C8—C9—H9	118.8	C19'—C21—H21E	109.5
O2—C10—C11	124.9 (2)	H21A—C21—H21E	104.7
O2—C10—C15	117.33 (19)	H21B—C21—H21E	37.6
C11—C10—C15	117.7 (2)	H21C—C21—H21E	76.7
C10—C11—C12	120.2 (2)	H21D—C21—H21E	109.5
C10—C11—H11	119.9	C19—C21—H21F	81.1
C12—C11—H11	119.9	C19'—C21—H21F	109.5
C13—C12—C11	121.6 (2)	H21A—C21—H21F	104.8
C13—C12—H12	119.2	H21B—C21—H21F	137.4
C11—C12—H12	119.2	H21C—C21—H21F	33.1
C12—C13—C14	120.9 (2)	H21D—C21—H21F	109.5
C12—C13—Cl2	120.29 (17)	H21E—C21—H21F	109.5
C14—C13—Cl2	118.76 (17)	O4—C22—C24	109.4 (2)
C16—C14—C15	117.36 (19)	O4—C22—C23	109.6 (2)
C16—C14—C13	126.0 (2)	C24—C22—C23	111.8 (2)
C15—C14—C13	116.59 (19)	O4—C22—H22	108.7
N2—C15—C14	122.49 (19)	C24—C22—H22	108.7
N2—C15—C10	114.65 (19)	C23—C22—H22	108.7
C14—C15—C10	122.86 (19)	C22—C23—H23A	109.5
C17—C16—C14	119.4 (2)	C22—C23—H23B	109.5
C17—C16—H16	120.3	H23A—C23—H23B	109.5
C14—C16—H16	120.3	C22—C23—H23C	109.5
C16—C17—C18	119.3 (2)	H23A—C23—H23C	109.5
C16—C17—H17	120.4	H23B—C23—H23C	109.5
C18—C17—H17	120.4	C22—C24—H24A	109.5
N2—C18—C17	122.9 (2)	C22—C24—H24B	109.5
N2—C18—H18	118.5	H24A—C24—H24B	109.5
C17—C18—H18	118.5	C22—C24—H24C	109.5
O3—C19—C20	108.7 (3)	H24A—C24—H24C	109.5
O3—C19—C21	109.4 (2)	H24B—C24—H24C	109.5
C20—C19—C21	115.5 (3)		
			
O3—Ti1—O1—C1	178.69 (16)	C4—C5—C6—N1	177.79 (19)
O4—Ti1—O1—C1	−76.91 (16)	C7—C5—C6—C1	177.9 (2)
O2—Ti1—O1—C1	49.2 (2)	C4—C5—C6—C1	−1.5 (3)
N2—Ti1—O1—C1	90.08 (16)	O1—C1—C6—N1	0.1 (3)
N1—Ti1—O1—C1	8.91 (15)	C2—C1—C6—N1	−179.7 (2)
O3—Ti1—O2—C10	−86.92 (16)	O1—C1—C6—C5	179.4 (2)
O4—Ti1—O2—C10	168.21 (15)	C2—C1—C6—C5	−0.4 (3)
O1—Ti1—O2—C10	41.3 (2)	C6—C5—C7—C8	1.1 (3)
N2—Ti1—O2—C10	−0.82 (15)	C4—C5—C7—C8	−179.5 (2)
N1—Ti1—O2—C10	80.46 (16)	C5—C7—C8—C9	1.2 (3)
O4—Ti1—O3—C19'	107.6 (14)	C6—N1—C9—C8	0.4 (3)
O1—Ti1—O3—C19'	−150.7 (14)	Ti1—N1—C9—C8	173.98 (17)
O2—Ti1—O3—C19'	8.3 (14)	C7—C8—C9—N1	−2.0 (4)
N2—Ti1—O3—C19'	−67.0 (14)	Ti1—O2—C10—C11	−179.65 (17)
N1—Ti1—O3—C19'	−103.0 (15)	Ti1—O2—C10—C15	0.5 (3)
O4—Ti1—O3—C19	−174.9 (3)	O2—C10—C11—C12	178.0 (2)
O1—Ti1—O3—C19	−73.2 (3)	C15—C10—C11—C12	−2.2 (3)
O2—Ti1—O3—C19	85.8 (3)	C10—C11—C12—C13	1.3 (3)
N2—Ti1—O3—C19	10.5 (3)	C11—C12—C13—C14	1.5 (3)
N1—Ti1—O3—C19	−25.5 (5)	C11—C12—C13—Cl2	−177.04 (17)
O3—Ti1—O4—C22	179.3 (3)	C12—C13—C14—C16	174.5 (2)
O1—Ti1—O4—C22	81.4 (3)	Cl2—C13—C14—C16	−6.9 (3)
O2—Ti1—O4—C22	−76.9 (3)	C12—C13—C14—C15	−3.3 (3)
N2—Ti1—O4—C22	−24.4 (5)	Cl2—C13—C14—C15	175.28 (16)
N1—Ti1—O4—C22	6.1 (3)	C18—N2—C15—C14	−3.2 (3)
O3—Ti1—N1—C9	128.4 (3)	Ti1—N2—C15—C14	179.36 (16)
O4—Ti1—N1—C9	−81.3 (2)	C18—N2—C15—C10	176.46 (19)
O1—Ti1—N1—C9	177.9 (2)	Ti1—N2—C15—C10	−1.0 (2)
O2—Ti1—N1—C9	15.1 (2)	C16—C14—C15—N2	4.1 (3)
N2—Ti1—N1—C9	91.8 (2)	C13—C14—C15—N2	−177.96 (19)
O3—Ti1—N1—C6	−57.6 (4)	C16—C14—C15—C10	−175.5 (2)
O4—Ti1—N1—C6	92.72 (15)	C13—C14—C15—C10	2.4 (3)
O1—Ti1—N1—C6	−8.15 (14)	O2—C10—C15—N2	0.4 (3)
O2—Ti1—N1—C6	−170.88 (15)	C11—C10—C15—N2	−179.40 (19)
N2—Ti1—N1—C6	−94.18 (14)	O2—C10—C15—C14	−179.95 (19)
O3—Ti1—N2—C18	−73.6 (2)	C11—C10—C15—C14	0.2 (3)
O4—Ti1—N2—C18	129.5 (3)	C15—C14—C16—C17	−1.6 (3)
O1—Ti1—N2—C18	21.80 (19)	C13—C14—C16—C17	−179.3 (2)
O2—Ti1—N2—C18	−176.1 (2)	C14—C16—C17—C18	−1.5 (3)
N1—Ti1—N2—C18	98.4 (2)	C15—N2—C18—C17	−0.2 (3)
O3—Ti1—N2—C15	103.48 (15)	Ti1—N2—C18—C17	176.69 (16)
O4—Ti1—N2—C15	−53.4 (4)	C16—C17—C18—N2	2.6 (3)
O1—Ti1—N2—C15	−161.11 (15)	C19'—O3—C19—C20	59.8 (5)
O2—Ti1—N2—C15	0.99 (14)	Ti1—O3—C19—C20	−95.6 (4)
N1—Ti1—N2—C15	−84.50 (14)	C19'—O3—C19—C21	−67.2 (5)
Ti1—O1—C1—C2	171.39 (17)	Ti1—O3—C19—C21	137.4 (2)
Ti1—O1—C1—C6	−8.4 (3)	C19—O3—C19'—C20	−64.4 (6)
O1—C1—C2—C3	−178.3 (2)	Ti1—O3—C19'—C20	32.8 (18)
C6—C1—C2—C3	1.5 (3)	C19—O3—C19'—C21	60.8 (5)
C1—C2—C3—C4	−0.7 (4)	Ti1—O3—C19'—C21	158.0 (10)
C2—C3—C4—C5	−1.3 (4)	O3—C19'—C20—C19	63.7 (5)
C2—C3—C4—Cl1	177.01 (18)	C21—C19'—C20—C19	−57.7 (5)
C3—C4—C5—C6	2.3 (3)	O3—C19—C20—C19'	−60.2 (5)
Cl1—C4—C5—C6	−176.04 (16)	C21—C19—C20—C19'	63.2 (5)
C3—C4—C5—C7	−177.1 (2)	O3—C19—C21—C19'	63.4 (5)
Cl1—C4—C5—C7	4.6 (3)	C20—C19—C21—C19'	−59.6 (5)
C9—N1—C6—C5	2.1 (3)	O3—C19'—C21—C19	−63.4 (5)
Ti1—N1—C6—C5	−172.73 (16)	C20—C19'—C21—C19	60.5 (5)
C9—N1—C6—C1	−178.61 (19)	Ti1—O4—C22—C24	150.1 (2)
Ti1—N1—C6—C1	6.6 (2)	Ti1—O4—C22—C23	−87.0 (3)
C7—C5—C6—N1	−2.8 (3)		
